# Simulation in Pre-departure Training for Residents Planning Clinical Work in a Low-Income Country

**DOI:** 10.5811/westjem.2015.9.28164

**Published:** 2015-11-18

**Authors:** Kevin R. Schwartz, Kimball A. Prentiss

**Affiliations:** * Massachusetts General Hospital, Harvard Medical School, Department of Pediatrics and Department of Emergency Medicine, Boston, Massachusetts; † Baystate Medical Center, Tufts University School of Medicine, Department of Emergency Medicine, Springfield, Massachusetts

## Abstract

**Introduction:**

Increasingly, pediatric and emergency medicine (EM) residents are pursuing clinical rotations in low-income countries. Optimal pre-departure preparation for such rotations has not yet been established. High-fidelity simulation represents a potentially effective modality for such preparation. This study was designed to assess whether a pre-departure high-fidelity medical simulation curriculum is effective in helping to prepare residents for clinical rotations in a low-income country.

**Methods:**

43 pediatric and EM residents planning clinical rotations in Liberia, West Africa, participated in a simulation-based curriculum focused on severe pediatric malaria and malnutrition and were then assessed by survey at three time points: pre-simulation, post-simulation, and after returning from work abroad.

**Results:**

Prior to simulation, 1/43 (2%) participants reported they were comfortable with the diagnosis and management of severe malnutrition; this increased to 30/42 (71%) after simulation and 24/31 (77%) after working abroad. Prior to simulation, 1/43 (2%) of residents reported comfort with the diagnosis and management of severe malaria; this increased to 26/42 (62%) after simulation and 28/31 (90%) after working abroad; 36/42 (86%) of residents agreed that a simulation-based global health curriculum is more useful than a didactic curriculum alone, and 41/42 (98%) felt a simulator-based curriculum should be offered to all residents planning a clinical trip to a low-income country.

**Conclusion:**

High-fidelity simulation is effective in increasing residents’ self-rated comfort in management of pediatric malaria and malnutrition and a majority of participating residents feel it should be included as a component of pre-departure training for all residents rotating clinically to low-income countries.

## INTRODUCTION

Pediatric and emergency medicine (EM) residents in the United States are increasingly interested in undertaking clinical rotations in low-income countries.^1^,^2^ In response to this interest, the number of residency programs offering opportunities to work clinically in low-income international sites has grown significantly, resulting in more U.S. residents rotating to these settings than ever before.^3^,^4^ The disease spectrum and available resources residents encounter in these settings are often vastly different from what they are accustomed to at their home training institutions. Thus, without additional training, residents may be ill prepared to function in these new environments. There are significant ethical considerations at stake in ensuring that U.S. residents working in low-income countries are competent in their practice there.^5^ Consequently, the need for high-quality, competency-based, pre-departure training to prepare residents for practice in these international settings has been recognized.^6^,^7^

Despite this, few formal pre-departure curricula presently exist to prepare residents for clinical work in low-income countries. 4 One survey of pediatric residency programs offering global health electives revealed that only 36% of programs offered any type of pre-departure training for their residents planning clinical work abroad. 8 Among those programs offering pre-departure training, there exists wide variability in the nature, duration and learning modalities employed in such training, and the optimal teaching methods for pre-departure training have not yet been established.^6^,^9^

At the same time, medical simulation is increasingly being used to help EM and pediatric residents gain knowledge, skills and comfort in situations they may have limited or no prior experience with, while allowing repetitive practice to occur in a safe environment without risk to patients.^10^,^11^

Simulation has a history of use in preparing learners to effectively manage stressful circumstances in foreign environments they have not previously encountered. It has been used effectively by the American military to prepare combat medics for the new and unfamiliar circumstances they encounter upon deployment abroad. 12 High-fidelity medical simulation is especially useful in pediatric EM given the relative rarity of severe childhood illness in the U.S. and other developed countries, limiting meaningful training opportunities for providers who care for acutely ill children.^13^,^14^ It has been used effectively to train practitioners to handle rare but critical pediatric emergencies.^10^,^13^,^14^ It follows that medical simulation has the potential to help residents preparing for clinical work in an unfamiliar, foreign country setting, managing pediatric diagnoses they have rarely, if ever, managed before.

To date, no pre-departure curriculum for residents planning clinical work in low-income countries has been published that incorporates the use of high-fidelity medical simulation. We hypothesized that a pre-departure global health curriculum incorporating the use of high-fidelity medical simulation would be effective in helping pediatric and EM residents improve comfort with specific pediatric emergencies commonly encountered during an international clinical elective in a low-income country yet uncommonly encountered within U.S. teaching hospitals.

## METHODS

### Simulation Curriculum Development

**Figure 1:**
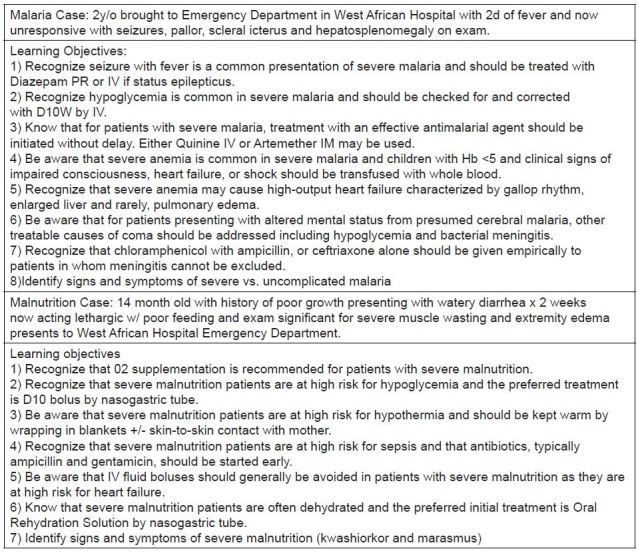
Clinical cases and learning objectives for simulations teaching residents how to treat malaria and pediatric malnutrition.

We developed two simulation cases for the pre-departure curriculum: a case requiring residents to diagnose and manage severe pediatric malaria and its sequelae and a case requiring residents to diagnose and manage severe pediatric malnutrition complicated by sepsis and hypoglycemia. These two topics were selected as the focus for the pre-departure simulation curriculum because of the relatively high frequency of emergent presentations of these diagnoses in West Africa, the region for which the pre-departure curriculum was designed, as well as the fact that most U.S.-trained residents were unlikely to have significant prior experience in managing these diagnoses at their home institutions. The simulation cases composing the curriculum and their respective learning objectives are described in Figure 1. We based learning objectives on World Health Organization (WHO) guidelines for acute treatment of severe malaria and severe malnutrition.^15^,^16^

We pre-programmed each simulation case using Laerdal SimBaby Scenario Editor software (Laerdal Medical Co, Wappingers Falls, New York), such that successful navigation of the case required performance of clinical tasks related to the learning objectives iterated in Figure 1. In addition to programming the cases, we created a content-standardized debriefing presentation on PowerPoint slides highlighting the learning objectives of each case.

### Participants

All participants in this curriculum were EM or pediatric residents planning a four- to eight-week clinical elective at the John F. Kennedy Medical Center in Liberia, West Africa, through the Health Education and Relief Through Teaching (HEARTT) non-governmental organization (NGO). HEARTT is an NGO facilitating placement of EM and pediatric residents from a variety of U.S. teaching hospitals into clinical rotations in the emergency department and inpatient wards of the John F. Kennedy Medical Center. HEARTT organizes an annual two-day pre-departure workshop for residents planning to rotate clinically in Liberia. Our simulation curriculum was integrated into this pre-departure workshop for two consecutive years. The institutional review board of Boston Medical Center approved this study.

### Procedure

Prior to participating in the simulation curriculum, all participants attended a 30-minute didactic lecture on malaria diagnosis and management and a 30-minute didactic lecture on malnutrition diagnosis and management. The content of these lectures reflected WHO guidelines regarding clinical management of these diagnoses. Participants were then given a five-minute orientation to the patient simulator and to the supplies and medications that would be available to them during the simulations. Next, participants were divided into teams of five to six residents. Each team participated in both of the simulations except where time constraints did not allow for this. When time did not allow all teams to participate in both simulations, as was the case for 23 participants, rather than participate in both simulations, each team would participate in one of the simulations and then watch the other simulation by closed circuit television. Irrespective of whether a team had participated in or watched a given simulation, they participated in the debriefing session following each simulation. Each simulation case ran for 10 minutes, followed by a 15-minute debriefing session.

For this program, the participants were restricted to using only those supplies and medications provided to them in the simulator room. These supplies and medications were selected based on what is actually available at the John F. Kennedy Medical Center in Liberia. The medications and supplies available were those typical of a regional hospital in West Africa. 17

### Program Evaluation

The primary outcomes evaluated in this study were participant self-rated comfort in the diagnosis and management of severe malnutrition and its complications and self-rated comfort in the diagnosis and management of malaria and its complications. Secondary outcomes included participants’ overall satisfaction with the curriculum, as well as learners’ opinions regarding usefulness and applicability of the curriculum to real world situations.

We evaluated all of the above outcomes by the administration of written surveys at three specific time points: A pre-simulation survey was administered to participants after they had attended didactic sessions but prior to participating in the simulation cases. Immediately after participating in the simulation curriculum, a second, post-simulation survey was administered containing the same questions as the pre-simulation survey and some additional questions regarding the experience of the simulation. Thereafter, between one and 11 months after completing the simulation curriculum, residents would complete a four- to eight-week clinical rotation in Liberia, West Africa at the John F. Kennedy Medical Center. Upon their return from this rotation, residents were asked to complete a third and final post-trip survey. Participants were randomly assigned a subject number upon enrollment and identified only by this number thereafter in order to maintain survey anonymity. Participants were provided a $10 Starbucks gift card for completing the surveys. Funding for these gift cards was provided from internal divisional funds from the division of pediatric emergency medicine at Boston Medical Center. There was no other funding for this study.

## RESULTS

Characteristics of curriculum participants are detailed in Table. Forty-three residents participated in this curriculum. Of these, 43 residents completed the pre-simulation survey, 42 of 43 (98%) completed the post-simulation survey, and 31 out of 43 (72%) completed the online post-trip survey. Residents travelled to Liberia from one month to 11 months after participating in the simulation curriculum with a median length of time elapsed between the simulation curriculum and their trip of five months.

Of those participating, 41/42 (98%) agreed with the statement “The simulations were a positive learning experience;” 36/42 (86%) agreed with the statement “A simulation-based global health curriculum is more useful than a didactic curriculum alone,” and 41/42 (98%) agreed with the statement “A simulator-based curriculum should be offered to all residents planning a clinical trip to a developing country.” Further, 28/31 (90%) residents agreed: “I was able to directly apply knowledge I learned in the simulator while abroad.”

**Figure 2:**
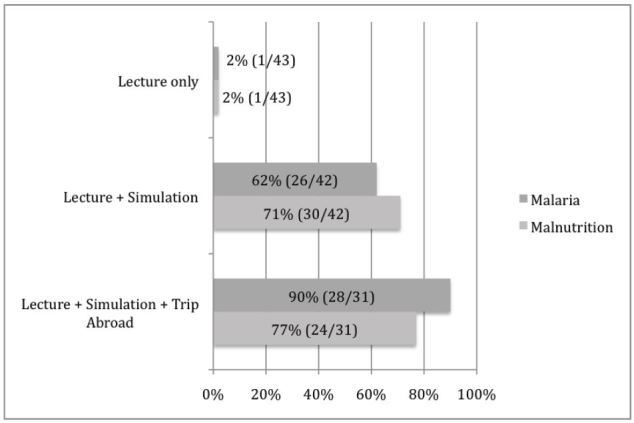
Proportion of residents agreeing with the statement “I am comfortable in the diagnosis and management of malaria (dark grey) or malnutrition (light grey) and their complications, surveyed at different time points.

The proportion of residents who agreed with the statement “I am comfortable with the diagnosis and management of severe malnutrition and its complications in children” after the didactic curriculum but prior to the simulation was 1/43(2%). After the simulation, 30/42 (71%) of participants agreed with this statement. After returning from clinical work in Liberia, 24/31(77%) agreed with this statement (Figure 2).

The proportion of residents who agreed with the statement “I am comfortable with the diagnosis and management of malaria and its complications in children” after the didactic curriculum but prior to the simulation was 1/43(2%). After the simulation 26/42 (62%) of participants agreed with this statement. After returning from clinical work in Liberia, 28/31(90%) agreed with this statement (Figure 2).

## DISCUSSION

Our study demonstrates that high-fidelity medical simulation improves pediatric and EM residents’ comfort in the diagnosis and management of pediatric malaria and severe malnutrition when incorporated into a pre-departure global health training. Malaria and malnutrition are both diagnoses U.S. residents are unlikely to have significant prior exposure to during their training, and yet the ability to manage these diseases competently while practicing in a setting such as West Africa or other malaria endemic area with high pediatric malnutrition rates is critical. The AAP Committee on Global Child Health has highlighted requisite preclinical training and adequate pre-trip preparation as critical components of a global health curriculum. 18 A survey of faculty directors of pediatric residency program global health tracks found that pre-trip preparation represents one of the most important strategies to alleviate the burden rotating residents place on global health partner institutions abroad. 9 Our study suggests that high-fidelity simulation should be a key part of high-quality pre-departure global health training with 98% of participants in our curriculum endorsing the statement that a simulator-based curriculum should be offered to all residents planning a clinical trip to a developing country.

While medical simulation has been used previously to successfully train clinicians in the management of malaria, such training was not targeted at improving comfort for practice in a low-income country setting. 19 To our knowledge, our curriculum is the first time simulation has been used to help residents prepare to treat this diagnosis as well as pediatric malnutrition and its sequelae in the low-income country hospital setting.

Our study limited groups of participants to teams of a maximum of six residents, which is, in general, the largest-sized team generally participating in other simulation studies. 13 A disadvantage to medical simulation is that there is a limit to the number of learners who can be in the simulator suite at any one time. In our study, due to time constraints, in some cases, not every resident was able to directly participate in both the malaria and the malnutrition simulation and some only had the opportunity to participate directly in one of the simulations while watching the other simulation by closed circuit television, an arrangement that has been used in other simulation studies. 20 Of note, when this subgroup was analyzed, the same benefit was observed in participants who watched a given simulation via closed circuit TV as for those who participated directly in the simulation, though the numbers were small and the study was not designed or powered to specifically address this question. Prior studies do corroborate this, however, and suggest that the learning benefit of simulation is not limited to those directly in the simulator room. 19 As residents’ time is often quite limited and there is much to cover during pre-departure training, the notion that watching a simulation can have similar benefit to direct participation may represent a means to engage a greater number of learners in simulation learning without requiring additional curriculum time.

Over half of the participants in our simulation curriculum reported prior clinical experience in a low-income country setting. Thus, many of the participants in our study may have already been familiar with the types of diagnoses this curriculum focused on, though we did not query the specific country in which they had worked and thus they may not have had direct prior experience with pediatric malnutrition and malaria.

The high-proportion of residents with prior experience in low-income country settings participating in our curriculum is unsurprising, as prior work has established that a major predictor of interest in clinical work in low-income countries in the presence of prior experience in such settings. 21 Therefore, it is likely that for any pre-departure curriculum, a sizable portion of participants will come to the curriculum with some degree of prior clinical experience in low-income country settings. Yet, even among participants with prior experience in low-income countries, the nature and location of such experience is highly variable and prior experience in a low-income country which is, for example, malaria endemic, compared to experience in a non-endemic country likely impacts comfort with this diagnosis. Furthermore, clinical experience in an observational capacity versus that in the capacity of a direct clinical care provider likely portends different levels of subsequent comfort with disease management. Even with prior direct experience with the diagnoses of malaria and malnutrition, residents who are training in the U.S. are infrequently encountering tropical medicine diagnoses in their everyday practice and comfort may decline over time. For such learners, a “refresher” experience in the simulator lab may be of very great value.

This project focused on acute presentations of disease in children. EM residents, in general, have less experience with treating children than pediatric residents. Pediatric residents, in general have less experience in treating medical emergencies as compared to EM residents. In subgroup analysis for our curriculum, there was no significant difference in the relative increase in self-rated comfort for pediatric as compared to EM residents, suggesting that both groups can benefit from increased practice in managing high acuity pediatric cases.

## LIMITATIONS

This study had several limitations. As the primary metric for assessment was learner self-rated comfort and knowledge as assessed by survey, it is not clear from our study that the increase in learners’ comfort regarding malaria and malnutrition management actually translates to changes in behavior. The question as to whether participation in this curriculum actually resulted in improved care and better outcomes for pediatric patients with malnutrition and malaria in Liberia was not directly addressed by this study. The surveys used to assess participants self-rated comfort and rating of the curriculum’s overall utility were not pre-validated which could have provided greater credulity to our survey’s findings.

Furthermore, our study did not include a control group that did not receive any high-fidelity simulator training for comparison. All residents participating in our pilot did receive a 30-minute didactic session on diagnosis and management of malaria and another 30-minute didactic on diagnosis and management of malnutrition. It was only after receiving these lectures that residents completed the first pre-simulation questionnaire. At that point, only 2% reported they were comfortable in the diagnosis and management of malnutrition and the diagnosis and management of malaria, respectively. Therefore, it seems that simulation is able to add considerably to a didactic curriculum. However, a control group who did not receive any simulator training at all was not employed in the study.

The simulation cases used in this study were created *de novo* by the authors using WHO guidelines for reference. Pre-existing, peer-reviewed simulation cases addressing the topics covered in this study were not publicly available. Such previously vetted simulation cases could potentially provide greater validity to the appropriateness and accuracy of the curriculum in mitigating the bias that exists in having the study authors also serve as the curriculum developers.

Future studies should examine the effect of simulation-based curricula on residents’ actual behaviors in their practice in international settings, as well as examine whether these behaviors indeed result in better outcomes for patients.

## CONCLUSION

The development of highly effective pre-departure clinical training for U.S. residents planning rotations in developing countries is critical. Our study found that residents planning clinical rotations in Liberia, West Africa, felt more comfortable in the management of pediatric malaria and malnutrition after a curriculum addressing these diagnoses that incorporated high-fidelity simulation. Participating residents felt strongly that simulation should be a part of pre-departure training offered to all residents. It is critical that we continue to study what type of pre-departure training best prepares residents for work abroad as we owe it to our global health partner sites to send residents who are well prepared to work in their setting, under proper supervision, and we are obliged to make sure our residents who are going to such settings feel as comfortable and safe as possible in their clinical practice there. Crucial to developing such curricula is knowledge of the available resources, common disease entities, setting-specific guidelines and standards of practice in the destination country. Low-income countries can vary greatly in the disease spectrum and available resources they have and selection of a pre-departure curriculum should take these differences into account.

Our study of a simulation-based pediatric malaria and malnutrition curriculum for residents planning clinical work in West Africa suggests that simulation has significant potential as an important component of pre-departure training for residents planning clinical work abroad.

## Figures and Tables

**Table:** Characteristics of curriculum participants.

Characteristic	Number of residents
Pediatric resident	17
Emergency medicine resident	26
Postgraduate year (PGY)	
PGY-1	0
PGY-2	2
PGY-3	37
PGY-4	4
Prior clinical work in a low-income country	
Yes	24
No	19
